# Postural Stability Characteristics of Transtibial Amputees Wearing Different Prosthetic Foot Types When Standing on Various Support Surfaces

**DOI:** 10.1155/2014/856279

**Published:** 2014-06-05

**Authors:** Nooranida Arifin, Noor Azuan Abu Osman, Sadeeq Ali, Hossein Gholizadeh, Wan Abu Bakar Wan Abas

**Affiliations:** Department of Biomedical Engineering, Faculty of Engineering, University of Malaya, 50603 Kuala Lumpur, Malaysia

## Abstract

*Purpose*. This study aimed to evaluate the effects of prosthetic foot types on the postural stability among transtibial amputees when standing on different support surfaces. *Materials and Methods*. The postural stability of 10 transtibial amputees wearing solid ankle cushion heel (SACH) foot, single-axis (SA) foot, and energy-saving and return (ESAR) foot was assessed. Results were compared with able-bodied participants. Anterior-posterior stability index (APSI), mediolateral stability index (MLSI), and overall stability index (OSI) were measured by computed posturography in an upright stance on firm, foam, and unstable support surfaces. *Results*. The mean OSI score of SACH foot was significantly lower than that of an ESAR foot when the participants were standing on a compliant surface. When compared to able-bodied group, MLSI score was significantly higher for each of the prosthetic foot groups while OSI score was significantly higher for ESAR foot only in foam condition. *Conclusions*. Differences between prosthetic foot types and groups (amputees versus able-bodied) can only be distinguished when individuals were standing on a compliant surface. Amputees exhibited an increased postural instability in the mediolateral direction than able-bodied individuals. Hence, the restoration of stability in the frontal plane and the enhancement of proprioception at the residual limb should be the basis of rehabilitation programs.

## 1. Introduction

Postural stability is achieved by maintaining an upright body alignment against gravitational force and preserving the equilibrium of the center of mass (CoM) in an individual's base of support [1]. Successful postural control requires the contribution from a complex sensory system comprising visual, somatosensory, and vestibular modalities as well as motor control systems [2, 3]. Healthy individuals greatly rely on somatosensory (70%), vestibular (20%), and visual (10%) perceptions when they stand on a firm surface under well-lit conditions [4]. By comparison, individuals mainly rely on vestibular and vision stimuli when the support surface changes because of inaccurate inputs from somatosensory components. Particularly, proprioception is one of the specialized components in the somatosensory system that provides information on the perceptions and awareness of joint movements and positions (passive and active) [5, 6]. Afferent inputs from mechanoreceptors located at the joints and muscles surrounding the ankle possibly influence the proprioceptive control of balance [7, 8]. Researchers suggested that the perception of the support conditions is necessary to retain the CoM within the support area in an erect human stance [9, 10]. As such, the ability to reorganize postural strategies depending on different support surface is a key to maintaining balance [9].

Ankle and hip strategies are considered responsible for the control of horizontal CoM movements in anterior-posterior and mediolateral directions, respectively [9, 11]. However, postural stability is decreased after individuals are subjected to below-knee amputation because of several factors, such as the lack of active ankle torques produced to restore balance in the sagittal plane, deficiency in weight shifting to control balance in the frontal plane, and distorted somatosensory inputs from the amputated side [12]. These factors can be explained by the loss of the biological ankle joint and a considerable amount of muscles in the lower leg, which functions as the source of proprioception in mobility and equilibrium [6]. As such, reduced proprioception is associated with asymmetry in weight bearing and decreased confidence of amputees [13]. Therefore, people with amputation are likely to refuse participation in daily and social activities because of a higher incidence of falling than able-bodied people [14].

For amputees to return to their daily life activities, the ability to maintain postural balance is essential while adapting to various support surface conditions. Balance can be relatively well managed in the comfort of an individual's house but may be very challenging when outdoor terrains are considered. For example, compliant (e.g., carpet, sand, and grass) or unstable surfaces reduce the ability to detect body orientation accurately [15]. Horak [16] also recommended that balance strategies on different support conditions should be evaluated in balance assessment to identify functional limitations and adaptation strategies of individuals with balance disorders. Previous studies on postural stability in unilateral below-knee amputees reported greater postural sway during quiet standing on a firm surface than able-bodied control subjects [17, 18]. Thus far, only one study has reported an increased level of body sway on a prosthetic leg compared with a sound leg during natural stance on a foam surface [19]. Considering previous studies, researchers suggested that the diversity in the mechanical designs of prosthetic feet is possibly one of the factors that contribute to unstable standing [11, 18].

Although prosthetic foot has been hypothesized to influence standing stability, knowledge about how or to what extent it controls stability remains unclear because of variations in the types of prosthetic feet tested in studies that may have determined the balance performance. Hence, it is vital for the clinician to understand the mechanism underlying integration of prosthesis limb into the balance system to compensate for the limb loss. This study aimed to determine the effect of different prosthetic foot types on the control of postural stability under various support surface conditions. This study was also designed to compare the results with those of able-bodied control subjects.

## 2. Materials and Methods

### 2.1. Participants

Using convenience sampling method, we enrolled 10 male unilateral below-knee amputees. All of the amputees were recruited from the University of Malaya Medical Centre rehabilitation clinics. Inclusion criteria were listed as follows: age > 20 years, unilateral below-knee amputation, with at least one year of experience in the current prosthesis, and able to walk without the use of an assistive device. Exclusion criteria for the amputees were described as follows: poor fittings of prosthesis and residuum pain. The participants with visual or vestibular impairment, lower limb musculoskeletal injury, and other neurological deficits were also excluded. A comparison control group consisting of nine male able-bodied participants were also included. This study was approved by the Institutional Ethics Committee Board, and written informed consent was obtained from each of the participants.

The subjective measure of prosthetic use and function was determined using a Houghton Scale questionnaire, which consists of four questions with a maximum possible score of 12 points [20]. To ensure similar balance status between amputees and able-bodied participants, we conducted the Berg Balance Test [21] and assessed functional balance performance. The test consists of 14 common daily tasks, such as sitting, standing, reaching, turning, and stepping. Each item is scored from 0 to 4 with a maximum score of 56 points: 0 to 20 indicates a high risk of falling; 21 to 40 indicates a medium risk of falling; and 41 to 56 indicates a low risk of falling. Subjects who failed to maintain equilibrium during the test were excluded from the study. All of the subjects completed SF12v2 to evaluate the health-related quality of life status of the participants [22].

### 2.2. Instrumentation and Procedures

The study employed a repetitive crossover study in which all of the amputees underwent a total of three testing sessions for three weeks. The control group was subjected to only one session for the completion of data collection. The amputees wore their corresponding prostheses that allowed the interchange of foot components. The same socket and suspension components were used throughout the study to eliminate any confounding effect of these variables. The amputees' current prosthetic sockets and components were optimally aligned using a laser liner before the assessment by the same registered prosthetist. The three prosthetic feet that were tested in this study included a solid ankle cushion heel (SACH) foot, a single-axis (SA) foot, and energy-saving and return (ESAR) foot Talux. All of the tested feet were prescribed according to the subject's foot size and body weight in addition to the activity level of the Talux foot. All subjects wore identical covered shoes and the same shoes were used in all the experiments.

The subjects familiarized themselves of the test procedures during their first visit. At the end of the first session, the prosthetic foot in the subject's prosthesis was exchanged for the first test foot. The subjects then returned to the laboratory to undergo postural stability assessment after one week of accommodation period [23]. The second test foot was subsequently attached to the prosthesis in the following week. The process was repeated until the subject had tested the third foot. Prosthetic foot and surface conditions were counterbalanced across subjects to negate order effects. In the last procedure, the test foot was replaced with the original foot.

### 2.3. Postural Balance Testing

Postural stability test was conducted using a Biodex stability system (BSS; Biodex Medical System, Shirley, NY, USA) for its known reliability in objective assessment of postural stability [24]. The system measures the degree of platform tilt about the anterior-posterior and mediolateral axes at a sampling rate of 20 Hz [25]. Platform stability, which ranged from 1 (least stable) to 12 (most stable), was varied in terms of spring resistance levels. BSS measures the overall stability index (OSI), anterior/posterior stability index (APSI), and medial/lateral stability index (MLSI), which represented the standard deviation of platform fluctuation from a horizontal position (zero point). Furthermore, OSI is considered as an efficient balance indicator of the ability to control balance [26]. The platform was integrated with computer software (Version 3.1 Biodex Medical Systems) that enables the device to calculate the stability indexes. An increased stability index during the assessment was interpreted as decreased postural stability. OSI, MLSI, and APSI scores were expressed as follows [25]:

(1) $$\begin{array}{c} \text{OSI}=\frac{\sqrt{\sum {\left(0-Y\right)}^{2}+\sum {\left(0-X\right)}^{2}}}{\text{number}\;\text{of}\;\text{samples}},\\ \text{APSI}=\frac{\sqrt{\sum {\left(0-Y\right)}^{2}}}{\text{number}\;\text{of}\;\text{samples}},\\ \text{MLSI}=\frac{\sqrt{\sum {\left(0-X\right)}^{2}}}{\text{number}\;\text{of}\;\text{samples}}, \end{array}$$

where *Y* is the total anterior-posterior deviation in the sagittal plane and *X* is the total medial-lateral deviation in the frontal plane.

Postural control was assessed under three different surface conditions: rigid, compliant, and unstable. For the rigid condition, the participants were asked to stand directly on a rigid and static platform. To simulate a compliant surface, we placed low-density polyethylene foam with a circular radius of 22 cm and a thickness of 2.5 cm on the platform [27]. Platform stability was then set at level 10 under an unstable condition. The subjects were instructed to step on the BSS platform and stand in a standardized position, in which each foot was positioned 17 cm between the heel centers and 14° between the long axes of the feet to eliminate between-subject variability during balance testing [28]. To ensure that this standardized position was maintained accurately for each test with all of the subjects, we marked and recorded the positions. During the test, the subjects were asked to keep their arms alongside the body and look straight ahead at a point on the wall approximately 1.5 m away at eye level to stabilize the head. All of the subjects stood on the platform for 20 s under all of the conditions. A mean score was calculated from the results of the three tests. The standard instruction “stand as still as possible” was given to all of the subjects to ensure consistency during assessment [29]. The subjects were allowed to rest for 30 s in a sitting position between trials and instructed not to change the position of their feet on the platform. Handrails could only be used to prevent falling if the subjects totally lost their balance. An assistant stood at the back of the subject for additional safety. Any trial with changes in foot position or balance loss was excluded.

### 2.4. Statistical Analysis

A total of 27 data sets from nine conditions (three support surface conditions and three prosthetic feet) for each of the stability indexes (OSI, APSI, and MLSI) were obtained. All of these data were initially screened to determine the normality of distribution and homogeneity of variance by using Shapiro-Wilk test. All of the data showed normal distribution. A 3 × 3 (support surface × prosthetic foot) repeated-measures analysis of variance (ANOVA) was used to examine the significance of differences between stability indexes. After the differences between groups were identified, post hoc HSD Tukey's test was applied to detect the specific area in which statistical differences were observed. Independent *t*-test was employed to compare the able-bodied control group with each prosthetic foot group. *P* ≤ 0.05 was considered significant. The effect size was also determined to indicate the significance of the results because of the small sample size used in this study. On the basis of Cohen's guidelines, we considered the effect size values > 0.14 to be significantly different [30]. Statistical analysis was performed using SPSS v16.0 (SPSS Inc., Chicago, IL, USA).

## 3. Results

The demographics summary of the participants is presented in Table 1. No significant differences were observed between the amputees and able-bodied group in terms of age, height, and body mass. The Berg Balance Score also showed no statistical difference between the groups.

The average values of OSI, APSI, and MLSI and the corresponding significant differences are shown in Table 2. When comparison was made between the prosthetic feet, our results showed that OSI was significantly higher in the ESAR foot than in the SACH foot (*P* = 0.04) when the subjects were standing on a foam surface compared with a firm and unstable support surface. A large effect size of 0.38 indicated that the differences were significant. Nevertheless, there was a noticeable trend of stability indexes being the lowest for SACH foot and the highest for ESAR foot in most of the conditions. Postural stability of the amputees as measured from the OSI, APSI, and MLSI indexes was not significantly affected by the interaction between prosthetic foot types and sensory conditions (*P* = 0.57, *P* = 0.08, and *P* = 0.66).

Although the stability indexes in prosthetic feet were higher than those of the able-bodied participants, significant differences were observed only in several conditions. For instance, the MLSI scores of the SACH foot and the SA foot were significantly higher than those of the able-bodied subjects (*P* = 0.05 and *P* = 0.03, resp.) when the subjects were standing on a foam surface. The OSI (*P* = 0.04) and MLSI (*P* = 0.04) of ESAR were significantly higher than those of able-bodied subjects standing on a foam surface. The effect size under all of the conditions was large (ranged from 0.20 to 0.39). No significant difference was evident in the APSI scores of the three prosthetic groups and the able-bodied group under all of the support surface conditions.

## 4. Discussion

This study investigated the effect of a prosthetic foot on the control of postural stability by comparing three types of prosthetic feet using the Biodex stability system. In addition, the importance of proprioception sensory information was examined by comparing the postural stability of able-bodied and below-knee amputee groups. Following amputation, complete loss of cutaneous, muscle, and joint receptors of the residual limb as well as distorted sensory feedback from the intact limb could affect postural stability [31]. However, the skin of the residual limb at the skin-socket interface, which has become more sensitive to the exerted pressure, possibly facilitates the movement of a prosthetic limb [32]. Hence, amputees should be able to control their prostheses to regulate the CoM in the support base to maintain stability during quiet standing.

Our results provided evidence that different prosthetic ankle mechanisms provided by various designs may influence postural stability in different support surface configurations. The stiffness of a prosthetic ankle has been proposed as the basis of stability in the unperturbed standing of amputees [33]. In particular, SACH, which provides no articulation at the ankle joint, likely minimizes the excursion of COP when individuals are standing on a compliant surface, thereby increasing the overall stability. For the ESAR foot, such as the Talux, the flexibility of the carbon fiber causes the body to exhibit larger excursion of COP and consequently reduces the overall stability of upright standing on a compliant surface. These findings further supported those of a previous study, in which a stiffer prosthetic foot may be used to enhance postural stability by decreasing body sway [11, 18]. In the three foot types, the control of stability in anterior-posterior and mediolateral positions is unlikely affected by different mechanisms on the ankle and support surface. No statistically significant difference was observed in the SA foot because of the control of the plantar flexion at the rear bumper, which is similar to the pretibial muscles of a normal foot [34]. Nevertheless, various contributing factors, such as restricted ankle mobility, weak hip abductor muscle strength, deficit in sensory organization, and low balance confidence [11, 13], have been linked to the altered balance conditions in amputees. To the best of our knowledge, this study is the first to investigate the effects of prosthetic foot types on the control of postural stability when subjects were standing on different support surface configurations.

In normal subjects, postural instability during quiet standing is resisted by muscle contraction to control ankle joint stiffness and counterbalance of the destabilizing gravitational torque in anterior-posterior and mediolateral directions [4]. For the amputees in this study, our findings suggested that postural stability requires more control in the mediolateral direction when standing on a compliant surface by utilizing the hip strategy. Moreover, increasing the use of hip musculature at the amputated limb was proposed as a strategy to receive more somatosensory inputs to compensate for the lack of sensory input due to amputation [35]. The ability to utilize the abductors and adductors of the hip possibly promotes an efficient weight transfer and prevents unnecessary compensation strategies, such as lateral trunk bending [36]. High postural instabilities in mediolateral directions can be used as an indicator of falling and confidence of amputees; with this information, amputees could understand their corresponding balance conditions and rehabilitation that they need to improve balance. Although significant difference was only observed under foam conditions, a decrease in postural stability of the amputees was showed when they were standing on firm or unstable surfaces compared with that of able-bodied participants.

We found that different postural stability characteristics can be determined between prosthetic foot types as well as between amputees and able-bodied groups when individuals are standing on a compliant surface. This was because the firm and flat surface provides accurate orientation information of body from the intact limb and residual limb while the compliant and tilting surface reduced the accuracy of information [37]. When standing on complaint surface, the normal ground reaction forces exerted at the feet were altered and this increased the movement of body's center of mass due to decreased effectiveness of the ankle to generate stabilisation torque to maintain equilibrium [38, 39].

Limitations in our study are acknowledged. We noted that the lack of significant differences under firm and unstable conditions may be caused by an increase in the dependence on other accurate sensory inputs from visual and vestibular systems. Therefore, future studies should occlude more than two sensory modalities for the differences between feet and groups to become apparent. Furthermore, the absence of prosthetic foot effect may be attributed to a less challenging nature of the task during quiet standing on firm and unstable platforms because the amputees were experienced and skilled prosthetic users. In addition, the small number of the subjects in this study may provide great differences between prosthetic feet and between groups, but such differences may not be statistically significant. Results in this study represent balance performance of transtibial amputees in general, which did not specifically distinguish between dysvascular and nondysvascular amputees. Although static balance has become an essential skill in rehabilitation process for the amputee populations to achieve independent standing and walking [17], further research should include stability assessment during walking and dual tasking.

## 5. Conclusions

The results suggest that prosthetic foot design affected the overall stability of below-knee amputees, particularly when subjects were standing on a compliant surface. Therefore, clinicians should consider this factor when prosthetic feet are prescribed to amputees who ambulate mostly on soft surfaces. Furthermore, amputees utilised the hip strategy to control postural stability in mediolateral directions in an upright stance on a compliant surface. These findings can be utilised to develop intervention during rehabilitation using different support surfaces which may lead to improvement in postural stability and reduce risk of falls in a person with lower limb amputations.

## Figures and Tables

**Table 1:** Clinical and demographic characteristics of participants.

Characteristics	Amputees	Able-bodied	*P* value
Mean ± SD age (years)	44.8 ± 13.5	44.1 ± 14.04	0.91
Mean ± SD height (m)	1.70 ± 0.06	1.66 ± 0.05	0.15
Mean ± SD weight (kg)	77.0 ± 17.9	73.9 ± 8.7	0.68
Sex (Male)	Ten	Nine	
Time after amputation (years)	7.1 ± 6.6		
Amputation cause	Five vascular, four trauma, and one tumor		
Mobility grade^†^	Five K2, Five K3		
Prosthetic foot	Seven single-axis		
	Three energy-saving-and-return		
Suspension	Three PTBs with pelite liner		
	Seven TSBs with pin lock		
Houghton Scale (mean ± SD)	10.5 ± 0.9		
BBS (total 56)	52.9 ± 4.9	56	0.08

^†^Based on Medicare K-level.

PTB: Patellar tendon bearing socket, TSB: Total surface bearing socket, and BBS: Berg Balance Score. Statistical significance of differences between the study groups was set as *P* < 0.05.

**Table 2:** The average and standard deviation of each prosthetic foot and control group during standing on different support surface configurations.

Groups	OSI			APSI			MLSI		
	Foam	Firm	Unstable	Foam	Firm	Unstable	Foam	Firm	Unstable
SACH^1^	1.88 (1.55)	1.71 (1.25)	2.01 (1.29)	0.95 (0.68)	1.08 (1.02)	1.24 (0.77)	1.31 (1.29)	1.09 (0.92)	1.35 (1.19)
SA^2^	2.28 (1.82)	1.9 (1.99)	1.81 (1.07)	1.26 (0.81)	0.80 (0.68)	1.09 (0.86)	1.68 (1.74)	1.58 (1.94)	1.22 (0.77)
ESAR^3^	2.55 (1.84)	1.86 (1.34)	2.29 (2.52)	1.48 (1.38)	0.65 (0.34)	1.12 (0.99)	1.88 (1.39)	1.59 (1.35)	1.82 (2.30)
Able-bodied^4^	1.13 (0.92)	1.1 (0.94)	1.52 (0.66)	1.02 (0.95)	0.91 (0.79)	1.11 (0.62)	0.33 (0.16)	0.49 (0.53)	0.76 (0.42)

Sig. two tailed (*P* ≤ 0.05)	1,3^§^						1,4*		
	3,4*						2,4*		
							3,4*		

*(1,4), (2,4), and (3,4) indicate significant difference between able-bodiedand prosthetic foot based on the independent samples *t*-test.

^§^(1,3) indicates significant difference between SACH and ESAR foot based on the post hoc analysis.
